# Brief Report: Trends in Incidence, Mortality, and Disability-Adjusted Life Years for Early-Onset Colorectal Cancer in Canada Between 1990 and 2019

**DOI:** 10.3390/curroncol31120571

**Published:** 2024-12-04

**Authors:** Igor Stukalin, Mehul Gupta, Katherine Buhler, Nauzer Forbes, Steven J. Heitman, Christopher Ma

**Affiliations:** 1 Division of Gastroenterology and Hepatology, Department of Medicine, University of Calgary, Calgary, AB T2N 4Z6, Canada; istukali@ucalgary.ca (I.S.);; 2 Division of Internal Medicine, Department of Medicine, University of Calgary, Calgary, AB T2N 4Z6, Canada; 3 Department of Community Health Sciences, University of Calgary, Calgary, AB T2N 4Z6, Canada

**Keywords:** early-onset colorectal cancer, incidence, mortality, disability-adjusted life years

## Abstract

Background: Colorectal cancer is the third most common malignancy globally. Early-onset colorectal cancer (EOCRC) is becoming a growing healthcare focus globally, particularly in North America. We estimated trends in incidence, mortality, and disability-adjusted life years (DALYs) for EOCRC in Canada between 1990 and 2019. Methods: We used the Global Burden of Diseases Study to evaluate trends in incidence, mortality, and DALYs for EOCRC in Canada between 1990 and 2019. Rates were estimated per 100,000 persons at risk with associated uncertainty intervals (UIs). Annual percentage changes (APC) were estimated using joinpoint regression with 95% confidence intervals (CIs). Results: In 2019, the incidence, mortality, and DALYs rates for EOCRC were 10.89 (95% UI 8.09, 14.34), 2.24 (95% UI 2.00, 2.51), and 111.37 (95% UI 99.34, 124.78) per 100,000 individuals, respectively. Incidence increased during the study period by 1.12%/year (95% CI 1.03%, 1.22%; *p* < 0.001). The largest increase in incidence in EOCRC occurred between 1990 and 2007, with an APC of 2.23% (95% CI 2.09%, 2.37%; *p* < 0.001). Mortality (APC 2.95%, 95% CI 1.89%, 4.02%; *p* < 0.001) and DALY (APC 2.96%, 95% CI 1.84%, 4.09%; *p* < 0.001) rates increased for males between 2001 and 2006. Conclusions: Our study reveals a substantial burden in EOCRC in Canada, with a significant increase in incidence.

## 1. Introduction

Despite advances in cancer prevention and population-based screening, colorectal cancer (CRC) remains the third most diagnosed malignancy globally [1]. CRC accounts for 10.2% of the global cancer incidence burden and 9.2% of all cancer-related deaths [2,3]. Recently, an alarming shift in CRC epidemiology was described, with a notable increase in the incidence of early-onset CRC (EOCRC) among individuals under the age of 50 [4,5,6]. EOCRC often presents at advanced stages and has a significantly worse prognosis than CRC diagnosed in older patients [7,8]. In an attempt to reduce long-term CRC mortality rates among younger patients, several jurisdictions, including the United States, lowered the age to start average-risk CRC screening from 50 to 45 years old [9]. Similar recommendations were not adopted in Canada. While the overall incidence of CRC declined in Canada, the rise in EOCRC poses unique challenges for gastroenterologists, colorectal surgeons, oncologists, and public health researchers [10].

Limited studies have investigated comprehensive population-based trends in EOCRC, specifically within the Canadian context, that can inform Canadian screening recommendations [11]. We evaluated trends and patterns in incidence, mortality, and disability-adjusted life years (DALYs) for EOCRC in Canada between 1990 and 2019, using data from the Global Burden of Disease Study (GBD).

## 2. Methods

Incidence, mortality, and DALYs attributable to EOCRC for the period between 1990 and 2019 were obtained from the GBD 2019 study, a global initiative that quantifies relevant epidemiological parameters for over 369 diseases in 204 countries using a variety of data sources and statistical methods [12]. EOCRC was defined as CRC diagnosed in patients between 15 and 49 years of age. Age-specific estimates of incidence, mortality, and DALY rates per 100,000 persons-at-risk and associated uncertainty intervals (UI) were obtained via pooling of estimates from constituent age ranges. Temporal trends in EOCRC were quantified using annual percent change (APC) with 95% confidence intervals (CI). Joinpoint regression employing a minimum of 2 years between consecutive inflection points and a maximum of 3 inflection points in total, as well as an *a priori* selected minimum APC difference worth detecting of 2.5%, was utilized to identify meaningful temporal trends in the Canadian burden of EOCRC without overfitting. Models were developed utilizing a grid search strategy, with model selection being performed based on a permutation test with 4499 interactions, with a significant threshold of *p* < 0.05. Joinpoint regression was conducted in Joinpoint software version 4.9.1.0 (Statistical Research and Applications Branch, National Cancer Institute, Rockville, MD, USA), and all other analysis and data visualization was performed in R, version 4.0.2 (R Group for Statistical Computing, https://cran.r-project.org, accessed on 1 June 2024).

## 3. Results

**Figure 1:**
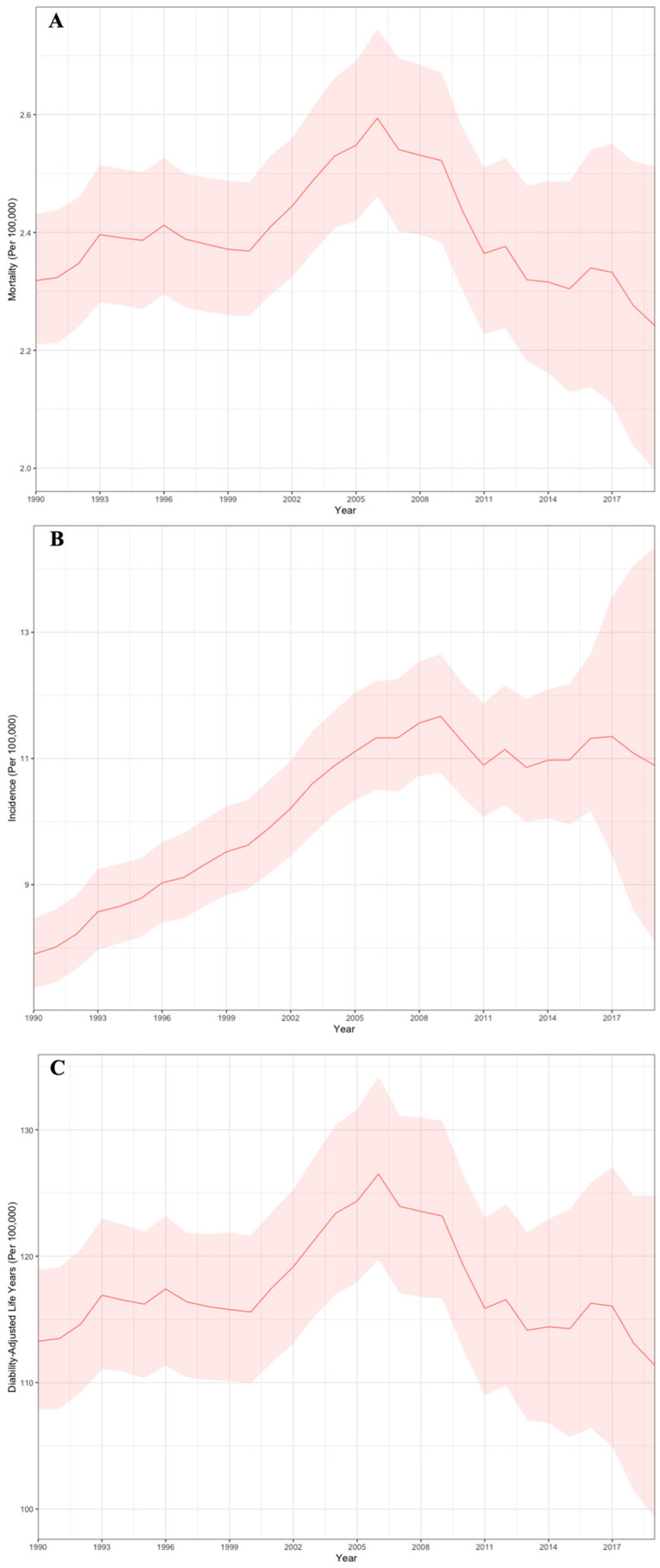
Mortality (**A**), incidence (**B**), and DALY rates (**C**) per 100,000 for EOCRC in Canada between 1990 and 2019.

In 2019, the total incidence rate of EOCRC was 10.89 (95% UI 8.09, 14.34) per 100,000 individuals, increased from 7.90 per 100,000 individuals (95% UI 7.36, 8.47) in 1990 (Figure 1). Analysis of temporal trends demonstrated that the most significant increase in the incidence of EOCRC in Canada occurred between 1990 and 2007 with an APC of 2.23% (95% CI 2.09%, 2.37%; *p* < 0.001). The most significant increase in incidence in men occurred between 2001 and 2005, with an APC of 5.03% (95% CI 2.65%, 7.46%; *p* < 0.001). Meanwhile, the largest increase in the incidence of EOCRC in females occurred between 1990 and 2002, with an APC of 2.29% (95% CI 2.10%, 2.48%; *p* < 0.001). The incidence of EOCRC decreased marginally between 2007 and 2019 (APC −0.42%, 95% CI −0.26%, −0.57%; *p* < 0.001).

The EOCRC mortality and DALY rates per 100,000 individuals in 2019 were 2.24 (95% UI 2.00, 2.51) and 111.37 (95% UI 99.34, 124.78), respectively. Overall temporal trends between 1990 and 2019 showed no significant change in EOCRC mortality (APC −0.06%, 95% CI −0.36%, 0.25%; *p* = 0.710) and DALY rates (APC −0.094%, 95% CI −0.34%, 0.16%; *p* = 0.46). EOCRC mortality (APC 2.95%, 95% CI 1.89%, 4.02%; *p* < 0.001) and DALY (APC 2.96%, 95% CI 1.84%, 4.09%; *p* < 0.001) increased for males between 2001 and 2006. 

The global EOCRC incidence, mortality, and DALY rates per 100,000 individuals in 2019 were 5.74 (95% UI 5.28, 6.27), 2.20 (95% UI 2.04, 2.37), and 108.25 (95% UI 100.20, 116.67). Global and United States APCs for incidence, mortality, and DALY rates for EOCRC between 1990 and 2019 are shown in Table 1.

## 4. Discussion

We estimated national trends in incidence, mortality, and DALYs for EOCRC between 1990 and 2019 in Canada and demonstrated several important findings. First, we confirmed an increase in EOCRC incidence. It is hypothesized that this rise in incidence may be attributable to environmental and lifestyle exposures, such as smoking, Westernized diet, obesity, and alcohol consumption [13,14,15]. Previous literature similarly identified male sex as a primary risk factor for EOCRC due to greater exposure to dietary, behavioral, and metabolic risk factors associated with cancer pathogenesis [16]. Our study shows that incidence rates of EOCRC stabilized and started to decrease slightly in Canada since 2007 [17]. However, compared to global estimates, the incidence of EOCRC still occurs at approximately twice the rate in Canada.

Second, our analysis demonstrates stability in mortality and DALY rates for EOCRC per 100,000 individuals in Canada. Whereas overall survival for CRC improved over time, a similar reduction in mortality was not demonstrated in EOCRC [18,19]. This may be explained by several factors. First, whereas effective population-based screening programs and better recognition of worrisome symptoms led to earlier stage diagnosis of CRC in older populations, EOCRC tends to be diagnosed at more advanced stages of the disease with resulting worse prognosis [20]. Second, the introduction of novel systemic treatments, such as targeted therapies and immunotherapies, improved advanced-stage CRC survival. However, this observation has not been similarly replicated in EOCRC [21,22]. A recent Canadian study identified that although patients with EOCRC are more likely to receive aggressive systemic and radiation therapy earlier in their disease course, their survival benefits are limited in comparison to older patients [23]. Looking forward, early detection and treatment of EOCRC may become more challenging as access to primary care in Canada becomes increasingly limited and endoscopy waitlists for routine CRC screening continue to grow [24].

Our study has several strengths. The GBD provides nationally representative estimates of the disease burden of EOCRC, and our study is the first analysis of incidence, mortality, and DALYs for EOCRC in Canada over time. Limitations include potential reporting bias and lack of individual-level data on risk factors for EOCRC within GBD, which provides population-level estimates based on administrative coding and Bayesian modeling. In conclusion, we highlight a substantial burden in EOCRC in Canada, underlining the importance of optimizing potential EOCRC screening regimens moving forward.

## Figures and Tables

**Table 1:** Annualized percent change with 95% confidence intervals (CI) for incidence, mortality, and DALY rates between 1990 and 2019.

	Canada		United States		Global	
	APC (95% CI)	*p*-Value	APC (95% CI)	*p*-Value	APC (95% CI)	*p*-Value
Incidence	1.1249 (1.0259, 1.2241)	<0.001	1.4959 (0.9961, 1.9982)	<0.001	1.7553 (1.5514, 1.9597)	<0.001
Mortality	−0.0578 (−0.3623, 0.2476)	0.710	0.6045 (0.2952, 0.9148)	<0.001	0.6396 (0.5263, 0.7531)	<0.001
DALYs	−0.0940 (−0.3430, 0.1556)	0.460	0.5769 (0.2539, 0.9011)	<0.001	0.5515 (0.4652, 0.6378)	<0.001

## Data Availability

Data are publicly available through the global burden of diseases study, which can be found at https://www.healthdata.org/research-analysis/gbd, accessed on 1 June 2024.
